# Drug–disease interactions in Swedish senior primary care patients were dominated by non-steroid anti-inflammatory drugs and hypertension – a population-based registry study

**DOI:** 10.1080/02813432.2020.1794396

**Published:** 2020-07-29

**Authors:** Katharina Schmidt-Mende, Morten Andersen, Björn Wettermark, Jan Hasselström

**Affiliations:** a Academic Primary Health Care Centre, Region Stockholm and Division of Family Medicine, Department of Neurobiology, Care Sciences and Society, Karolinska Institute, Sweden; b Department of Drug Design and Pharmacology, Faculty of Health and Medical Sciences, University of Copenhagen, Copenhagen, Denmark; c Department of Pharmacy, Disciplinary Domain of Medicine and Pharmacy, Uppsala University, Uppsala, Sweden

**Keywords:** Primary health care, aged, inappropriate prescribing, drug–disease interactions, cross-sectional studies, pharmacoepidemiology

## Abstract

**Objective:**

Drug–disease interactions (DDSIs) are present when a drug prescribed for one disease worsens a concomitant disease. The prevalence of DDSIs in older patients in primary care is largely unknown, as well as to what extent physicians individualize drug prescribing in relation to concomitant diseases. We therefore analysed the prevalence of DDSIs in older patients in primary care and explored to what extent physicians take possible DDSIs into account when prescribing.

**Design and Setting:** Cross-sectional population-based register study in primary care in Region Stockholm, Sweden. Thirty-one DDSIs derived from Irish STOPP-START-Criteria were assessed. We derived data from a regional administrative healthcare database including information on all healthcare consultations and dispensed prescription drugs in the region. Data on demography, diagnoses, drug dispensations and healthcare consumption were extracted. Drugs were assessed during 2016.

**Subjects:**

A total of 336,295 patients aged ≥65 registered with one of the 206 primary care practices in Region Stockholm.

**Main outcome measures:**

Prevalence and prevalence differences for DDSIs.

**Results:**

In 10.8% of older patients, at least one DDSI was observed. Non-steroidal anti-inflammatory drugs (NSAIDs) were implicated in more than 75% of cases. The most common DDSI was NSAID/hypertension (8.1%), followed by NSAID/cardiovascular disease and loop diuretics/urinary incontinence (both 0.7%). The use of NSAIDs among patients with heart failure or impaired renal function was 15% lower than among patients without these diseases.

**Conclusion:**

DDSIs were present in every tenth older patient in primary care. Patients with cardiovascular disease receive NSAIDs to a lower extent, possibly indicating physician awareness of DDSI.

**Key points**
- Evidence on the prevalence of drug–disease interactions in older patients in primary care is sparse despite their potential to cause harm.
- In this study, we found that every 10th older patient attending primary care had at least one drug–disease interaction.
- Interactions with NSAIDs were far more common than interactions with other drugs.
- The use of NSAIDs among patients with heart failure or impaired renal function was 15% lower than among patients without these diseases.

## Introduction

Older people are a growing population group and WHO foresees every fourth person to be 65 or older by 2050 [1]. Parallel with age, the number of diseases increase. Multimorbidity, defined as the presence of two or more chronic diseases, is present in more than 50% of older patients in a primary care setting [2,3]. Multimorbidity patients are at risk to receive drugs that treat one condition (=indication for drug treatment) but at the same time worsen a comorbidity (=interacting disease) [4]. One example of such a drug–disease interaction (DDSI) is the use of non-steroidal anti-inflammatory drugs (NSAIDs) against osteoarthritic pain in a patient who has heart failure.

There is no universally applicable list of interacting drugs and diseases. Most criteria for potentially inappropriate prescribing do not include DDSIs as a specific category [5].

The assessment of the frequency and nature of DDSIs in a primary care setting is important as they may have deleterious effects for older patients [6]. Primary care has a key role in the care of multimorbid older patients [7] and the majority of drugs to this patient group are prescribed by primary care physicians [8]. There is limited knowledge on the prevalence and risk factors for DDSIs in older patients attending primary care. We are aware of one systematic review from 2013 including eight studies [9] as well as five studies published since 2013 [10–14] on the prevalence of DDSIs. Ten of these 13 studies were conducted in the United States, one in China [14], and two in Europe [12,13]. Only one study was performed in European primary care [13]. As definitions of DDSIs and settings vary, large differences in prevalence are found, ranging from 3% [15] to 50% [11]. Studies in nursing home residents [11] report higher prevalence (50%) than studies in community-dwelling older people (15–20%) [10,12].

Our aim was to explore the prevalence of DDSIs in older patients attending primary care in a European capital (Stockholm, Sweden). We hypothesised that the prevalence of interacting drugs is lower in patients with interacting disease than in patients without.

## Material and methods

### Study design and setting

This was a retrospective, cross-sectional population-based register study. All patients aged ≥65 on 1 January 2016, registered with any of the totally 206 primary healthcare practices in the Region of Stockholm, Sweden, in December 2015, alive 31 December 2016, and resident during the entire year 2016 were included. Only ≈1% of the community-living population in Stockholm aged ≥65 was not registered with a primary care practice and thus not included in the analyses. Of note, nursing home residents (*n* ≈ 15.000 corresponding to ≈4% of the population aged ≥ 65 in 2016 in Region Stockholm) were not analyzed as they receive medical care from geriatricians instead of primary care.

### Swedish primary care system

In Sweden, primary healthcare is provided by public and privately run primary healthcare practices that operate under contracts with the county council, the public payer for healthcare for all citizens in a defined geographic area [16]. Patients may seek specialist care without a referral from a GP or other primary health care professional, as primary care does not have a gatekeeper function [17]. However, when people experience a health problem, their first point of contact is typically a GP or nurse at a primary care practice. People aged 65 years and older in Region Stockholm meet their GP a mean of 3.8 times a year. All residents have access to health care for low patient fees, and for people over the age of 85 there is no patient fee at all. Health expenditures as well as medicine costs are covered by the national insurance system. With exception for some private caregivers, the majority (>90%) of prescribing physicians in Region Stockholm have access to the patient´s entire drug list as they share the health record. Dispensing pharmacies have access to the entire list of prescribed drugs independent of the prescriber.

### Data source and variables

Data were extracted from Stockholm regional healthcare data warehouse (Swedish VAL) [16,17], which is a database that contains all data on all health care consumption, diagnoses, dispensed drug prescriptions, migration, and deaths for all 2.4 million inhabitants of Region Stockholm. Data recorded in VAL is the basis for reimbursement and follow-up monitoring of supplied healthcare for all citizens in the region. ICD-10 diagnoses recorded during hospital stays are the same as for the National Patient Register [18]. With some exceptions (for example, cancer), diagnoses in the National Patient Register have a positive predictive value of 85% to 95%, which implies high validity [21]. VAL even includes diagnoses recorded at primary care consultations. Of note, diagnoses given by some private caregivers who do not have contract with the public payer as well as diagnoses from caregivers outside Region Stockholm are not recorded in VAL. Still, as the majority of patients consulting those caregivers even consult primary care and hospitals in Region Stockholm we estimate the risk for missing data to be low (<5%). Information on at least one diagnosis was available for more than 95% of primary care consultations, for 99% of specialist ambulatory consultations, and for 99% of hospitalizations. Dispensed prescription drugs in VAL are derived from the Swedish prescribed Drug Register [19]. The validity of this register is high [20], more than 99% of prescriptions are registered with unique identifiers. Of note, drugs given during hospital stays and over-the-counter drugs are not included. We extracted information on sex, age, migration, death, ICD-10 diagnoses at consultation in primary care, specialist ambulatory care and hospitalization, and dispensed prescription drugs regardless of prescriber category.

### Definition of drug–disease interactions

DDSIs were extracted from the STOPP-section of Irish STOPP-START criteria version 2 [5,21]. However, they do not contain a separate list of DDSIs. Therefore, we selected 29 from a total of 80 STOPP-indicators if they werea DDSI according to the definition provided by Pugh et al. [4] (Appendix Table 1). Some of the STOPP-indicators contained not only one but several (2 to 5) DDSIs, for example D1 (‘tricyclic antidepressants with dementia, narrow angle glaucoma, cardiac conduction abnormalities, prostatism, or prior history of urinary retention (risk of worsening these conditions)’). The final list comprised 31 DDSIs (Table 1). Patients were considered to have a DDSI when they had an ICD-10-code for the interacting disease (Table 1) and had purchased at least one prescription of the interacting drug(s) in 2016. We analysed drugs that had been dispensed after a prescription by a physician working in primary care, specialist ambulatory or inpatient care in Sweden, but not over-the-counter drugs or drugs given during hospital stay. The drugs were classified in accordance with the Anatomical Therapeutic and Chemical (ATC) classification system [22]. Dermatologicals (ATC group ‘D’) were excluded from analyses.

**Table 1.** 31 drug–disease interactions that were assessed for their prevalence.

STOPP- criteria	Interacting drug	ATC	Interacting disease	ICD-10 and assessment period
B8	Thiazide diuretic	C03A	gout	M10 ≥once 2012–2016
B9	Loop diuretic	C03CA	urinary incontinence	N39.3, N39.4 ≥once 2012–2016
C2	(Low-dose) acetylsalicylic acid without proton-pump-inhibitor	B01AC06, A02BC acetylsalicylic acid ≥ once 2016 but no proton-pump-inhibitor 2016	peptic ulcer disease	K25-28 ≥once 2015–2016
C2	Acetylsalicylic acid without proton-pump-inhibitor	N02BA, A02BC acetylsalicylic acid ≥ once 2016 but no proton-pump-inhibitor 2016	peptic ulcer disease	K25-28 ≥once 2015–2016
D1	Tricyclic antidepressants	N06AA	dementia	F00-03, G30 ≥once 2012–2016
D1	Tricyclic antidepressants	N06AA	narrow angle glaucoma	H40.2 ≥once 2012–2016
D1	Tricyclic antidepressants	N06AA	benign prostate hyperplasia	N40 ≥once 2012–2016
D3	Neuroleptics with moderate-marked antimuscarinic/anticholinergic effects	N05AH02, N05AF01, N05AB02, N05AF05	benign prostate hyperplasia	N40 ≥once 2012–2016
D6	Antipsychotics (i.e. other than quetiapine or clozapine)	N05AA, N05AB, N05AD, N05AE, N05AF	M Parkinson	G20 ≥once 2012–2016
D8	Anticholinergic drugs	A03AB, A03BA, A03BB, A04AD, C01BA,N02AG, N04A, N05AA, N05AF03, N05AH02, N05BB01, N06AA, R06AA02, R06AB, R06AD, R06AX02	dementia	F00-03, G30 ≥once 2012–2016
E4	NSAIDs	M01A without M01AX05	impaired renal function	N18.3-18.5, N18.9 N19.9 ≥once 2012–2016
F1	Prochlorperazine or metoclopramide	A03FA01	M Parkinson	G20 ≥once 2012–2016
F3	Anticholinergic/antimuscarinic drugs	A03AB, A03BA, A03BB, A04AD, C01BA,N02AG, N04A, N05AA, N05AF03, N05AH02, N05BB01, N06AA, R06AA02, R06AB, R06AD, R06AX02, G04BD	constipation	K59 ≥once 2015 AND ≥ once 2016
F3	Oral iron	B03A	constipation	K59 ≥once 2015 AND ≥ once 2016
F3	Opioids	N02A	constipation	K59 ≥once 2015 AND ≥ once 2016
F3	Verapamil	C08DA01	constipation	K59 ≥once 2015 AND ≥ once 2016
F3	Aluminium antacids	A02AD01	constipation	K59 ≥once 2015 AND ≥ once 2016
G3	Anti-muscarinic bronchodilators	R03BB01, R03BB04	narrow angle glaucoma	H40.2 ≥once 2012–2016
G3	Anti-muscarinic bronchodilators	R03BB01, R03BB04	benign prostate hyperplasia	N40 ≥once 2012–2016
G4	Benzodiazepines	N05BA, N05BA01, N05CD02, N05CD03	obstructive sleep apnea	G47.3 ≥once 2012–2016
H1	Non-COX-2 selective NSAIDs without proton-pump-inhibitor or H2-blockers	M01AB, M01AC, M01AE, M01AX01	peptic ulcer disease	K25-28 ≥once 2015–2016
H2	NSAIDs	M01A without M01AX05	hypertension	I10-15 ≥once 2012–2016
H2	NSAIDs	M01A without M01AX05	heart failure	I50 ≥once 2012–2016
H7	COX-2 selective NSAIDs	M01AH, M01AB05	concurrent cardiovascular disease (coronary heart disease, peripheral arterial disease, cerebrovascular disease, TIA)	I20-25, I70-79, I63.0-I63.9, I61.0-I61.9, I64, G45.9, Z86.6A ≥ once 2012–2016
H9	Oral bisphosphonates	M05BA, M05BB	history of upper gastrointestinal disease i.e. dysphagia, oesophagitis, gastritis, duodenitis, or peptic ulcer disease, or upper gastrointestinal bleeding	K20-K29, K31 ≥once 2015 − 2016
I1	Antimuscarinic drugs	G04BD	dementia	F00-03, G30 ≥once 2012–2016
I1	Antimuscarinic drugs	G04BD	narrow-angle glaucoma	H40.2 ≥once 2012–2016
I1	Antimuscarinic drugs	G04BD	benign prostate hyperplasia	N40 ≥once 2012–2016
J2	Thiazolidenediones	A10BG	heart failure	I50 ≥once 2012–2016
J4	Oestrogens	G03C (only oral)	breast cancer	C50 ≥once 2012–2016
J4	Oestrogens	G03C (only oral)	venous thromboembolism	I26, I82 ≥once 2012–2016

### Definition of diseases

Diseases were assessed based on ICD-10 codes registered in primary care, specialist ambulatory or inpatient care.

**Figure 1.**
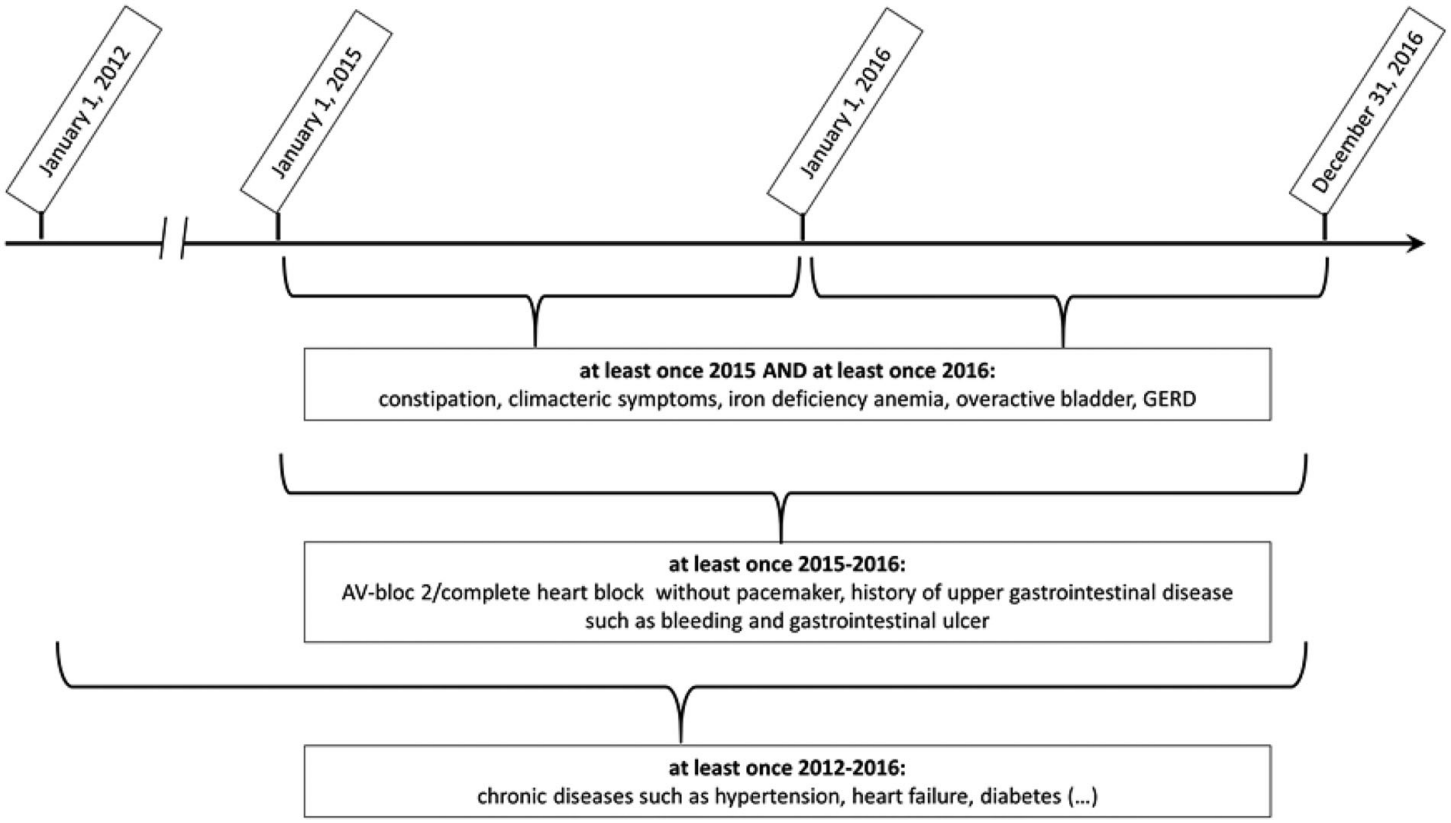
Time frames for assessment of diseases. GERD: Gastroesophageal reflux disease

We differentiated between (1) chronic diseases that described the study population, identified by ≥one code 2012–2016: cardiovascular disease (hypertension, coronary heart disease, heart failure, peripheral arterial disease, atrial fibrillation), cerebrovascular disease (stroke, transient ischemic attack), cancer, chronic respiratory disease (chronic obstructive pulmonary disease, asthma), diabetes; (2) interacting diseases (diseases that are potentially worsened by the interacting drug). Depending on the severity and chronicity of disease, we applied three different time frames (Figure 1): (a) For chronic diseases: ≥one code 2012–2016; (b) For severe but less chronic disease: ≥ one code 2015–2016; (c) For less severe disease: ≥one code 2015 and 2016; and (3) a pain-diagnosis in order to identify patients with possibly contraindicated NSAIDs: osteoarthritis (M15-19) or rheumatologic disease (M05-14) ≥once 2012–2016 or the same chronic pain diagnoses (pain in shoulder, leg, foot, back, fibromyalgia M75-77, M79, M53, M54, R52 without R52.0, migraine G43, headache G44, R51) ≥once 2015 and ≥once 2016.

### Other variables

Demographic variables included sex and age on 1 January 2016, as well as death and migration in or out of Region Stockholm in 2016. Regarding healthcare consumption, the following variables were assessed: registration with a primary care practice in December 2015, physician visits in primary health care, and visits at emergency departments at one of the total of seven emergency hospitals in Region Stockholm.

In order to calculate the total number of drugs prescribed to a patient we assessed the number of dispensations during a 4-month period (1 January 2016 to 30 April 2016). The choice of a 4-month interval is due to Swedish reimbursement regulations: Each prescription generally mandate a time window of three months between two dispensations [23].

### Statistics

We calculated prevalence and two-tailed 95% binomial confidence intervals as well as prevalence differences and two-tailed 95% confidence intervals for diseases and DDSIs. For numerical values we present relevant descriptive statistics, mean and standard deviation (SD) or median and interquartile range (IQR).

Data extraction was performed with SAS Enterprise Guide version 7.1, and statistical analyses were undertaken in STATA version 14.

## Results

### Study population

After the exclusion of 16,648 people (death and/or move), the study population consisted of 336,295 patients with a mean age of 74.3 years (SD 7.4). There were more women than men (55 vs. 45%) (Table 2).

**Table 2.** Basic characteristics of study population (*n* = 336,295).

	Total	% (if not indicated otherwise)
Age in years	65–74	59.9
	75–84	28.6
	85 and older	11.6
Morbidity^a^	Cardiovascular disease^b^	59.4
	Cancer	17.8
	Diabetes	15.4
	Chronic respiratory diseases^c^	12.2
	Cerebrovascular disease^d^	7.4
Healthcare consumption	Median nr of visits with GP (IQR)	4 (2;9)
	Median nr of emergency department visits (IQR)	0 (0;1)

^a^ 1 January 2012 to 31 December 2016.

^b^ Hypertension, coronary heart disease, heart failure, atrial fibrillation, peripheral arterial disease.

^c^ Chronic obstructive pulmonary disease, asthma.

^d^ Stroke, transient ischemic attack.

Cardiovascular diseases were the most prevalent disease group. Individuals met their GP in median 4 times a year. Each patient was dispensed a median of four (IQR 2;7) prescription drugs. Of note, 16% had no drug treatment at all, and 12% had ≥10 drugs.

### Prevalence of drug–disease interactions

In 10.8% of patients, at least one DDSI was seen. Nine per cent (*n* = 37,773) of the study population had one DDSI, 1.4% had two DDSIs, and <0.4% had three or more DDSIs.

**Figure 2.**
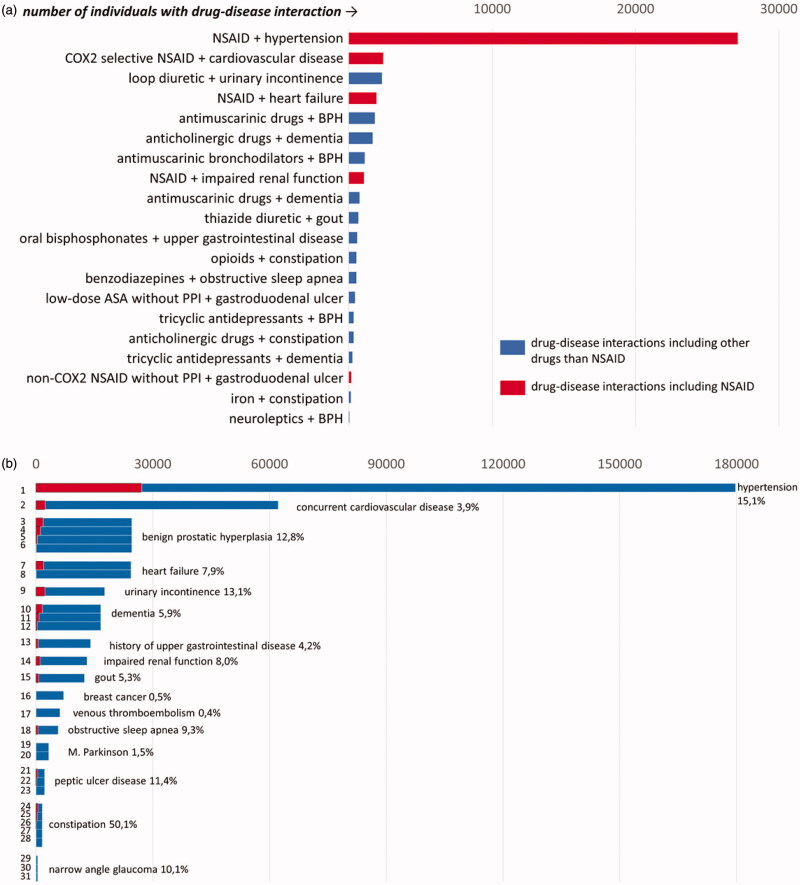
(a,b) Number of patients with drug-disease interaction in the total population (2a) and grouped by interacting disease (2 b) (total population *n* = 336,295). For corresponding numbers: see appendix, Tables 2 and 3. (a) Prevalence of the 20 most commonly seen drug-disease interactions.

Figure 2(a) shows the 20 most common DDSIs. One DDSI was far more common than all others: use of NSAIDs in patients with hypertension. It was observed in 8.1% of total study population, thus explaining 75% of the total prevalence of DDSIs. Moreover, NSAIDs were included in five out of the 20 most commonly observed DDSIs shown in Figure 2(a) (red bars). It is important to note that there was a long tail of 11 DDSIs that were very uncommon and prescribed each to less than 100 patients (Appendix Table 2).

**Figure 3.**
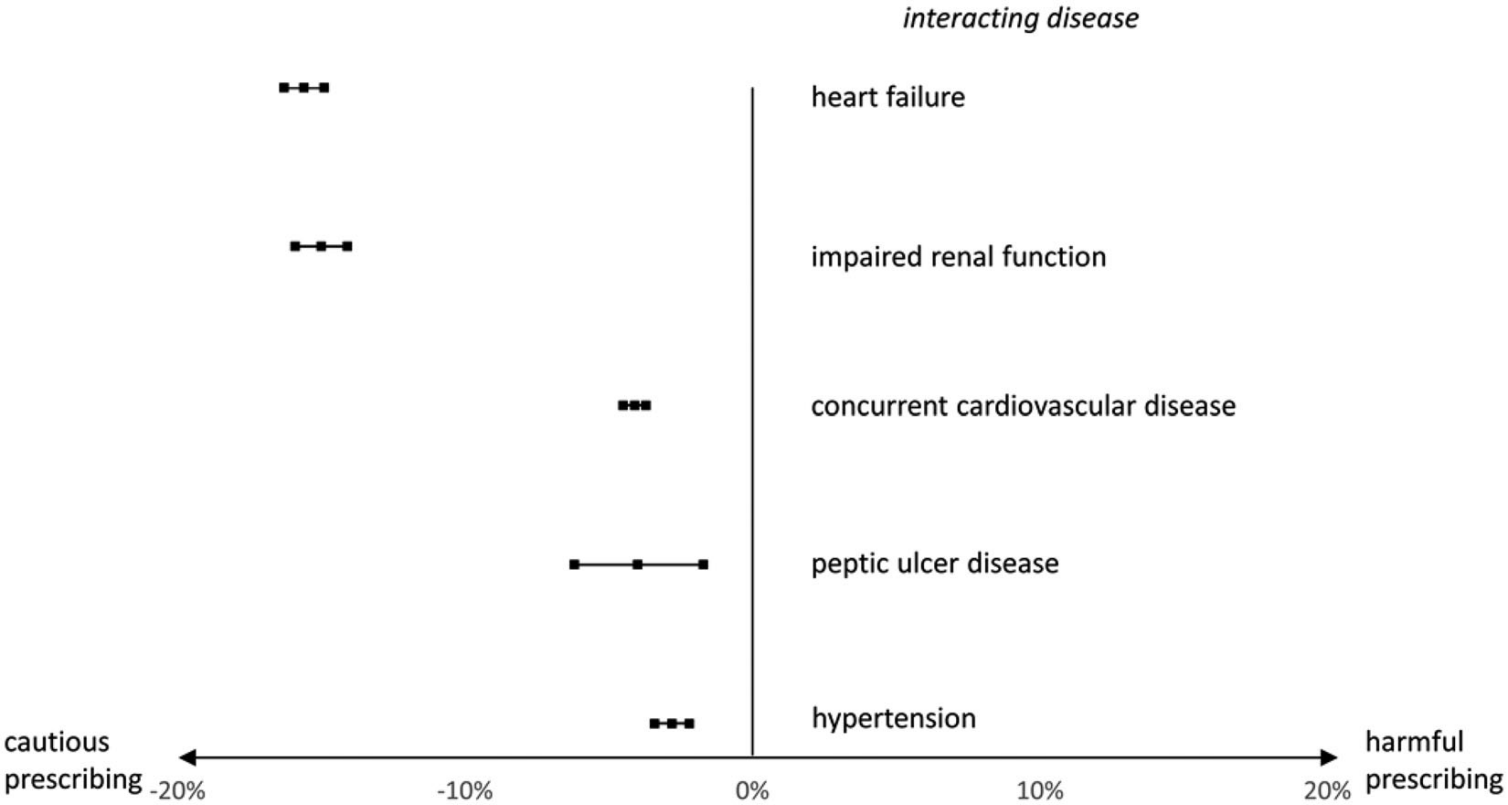
Prevalence differences and 95% confidence intervals of NSAID use in patients with a pain diagnosis and with/without an interacting disease.

There were a total of 31 DDSIs in relation to 17 interacting diseases. The most common interacting disease was hypertension (Figure 2(b) and Appendix Table 3).

Several interacting diseases such as constipation and benign prostate hyperplasia were potentially worsened by several interacting drugs. The prevalence of the interacting drug in patients with an interacting disease was at its most 50% (drugs interacting with constipation). Of note, this high percentage was mainly due to opioids (30%) (Appendix Table 3).

### Prevalence of NSAIDs in patients with or without interacting disease

NSAIDs were far more common than all other interacting drugs, and were implicated in five of the 20 most prevalent DDSIs. The prevalence of NSAID in two different patient groups having a chronic pain diagnosis was compared (Appendix Figure 1). We found that NSAID use was less prevalent in patients with an interacting disease/disease group compared to patients without (Figure 3 and Appendix Table 4). For example, within the group of patients with chronic pain, those with heart failure had a 15% lower risk of NSAID use compared to patients without heart failure (13 vs. 28%). The corresponding numbers for impaired renal function were 12 vs. 27%, for concurrent cardiovascular disease 7 vs. 11%, for peptic ulcer disease 13 vs. 17%, and for hypertension 25 vs. 28%.

## Discussion

### Summary

In a total primary care population in Region Stockholm, 10.8% of older patients were found to have at least one DDSI. The most common interacting drugs were NSAIDs which were implicated in five DDSIs and accounted for more than 80% of potentially inappropriate prescribing. At most 50% of patients with an interacting disease were treated with an interacting drug. Among patients with pain diagnosis, NSAIDs were prescribed to a lower extent to those with interacting disease compared to those without interacting disease.

### Strengths and limitations

To our knowledge, this is the largest population-based analysis on the prevalence of DDSIs in older patients attending primary care. DDSIs were selected from and defined in relation to STOPP-START criteria [5]. These criteria as a whole have high clinical relevance and predictive ability [21]. As the area of DDSI suffers from incomplete definitions both as regards which interactions to use (for example, STOPP-START criteria [5], Beers criteria [24]) as well as which time frames to use when choosing diagnoses and drug use it may be hard to judge whether our findings are due to the criteria used or reflect a true Swedish prescribing situation. We have described the selection process in detail in the appendix, making it possible to critically evaluate the basis for our choices. The reader should also be aware that the definition we chose for DDSIs is not a fully validated one, and there may be more DDSIs than the ones included in the STOPP-START criteria. For example, the use of cortisone in patients with diabetes is not included as STOPP-indicator. It is therefore likely that the true prevalence of DDSIs is higher than we reported.

Register data are easily collected and allow to analyze large populations without recall or selection bias. There are however possible bias stemming from the validity of diagnoses. Diagnoses drawn from hospital-based registers usually have high validity [18] as have common diagnoses like hypertension, diabetes [25] and heart failure [26] in primary care. Still, the validity of diagnoses coded in primary care has not been sufficiently analysed. In patients with renal failure, underreporting of ICD-10 diagnoses is a bigger problem than overreporting [27]: 25% of patients aged 65 and older where a creatinine had been taken had renal impairment stage 3 or 4, whereas only 5% had an ICD-10 code for renal impairment. We therefore think that the prevalence of interacting diseases and thus the true prevalence of DDSIs is higher than the one we reported due to insufficient coding of ICD-10 diagnoses. More uncommon diagnoses such as glaucoma though, have been less studied.

Although the validity of the prescribed drug register from which we collected our dispensation data is high [19], information on intake of drugs rather than dispensation is a limitation. Five DDSIs could moreover not be assessed due to lack of clinical data, such as STOPP-indicator I1 (antimuscarinic drugs for overactive bladder syndrome with concurrent chronic cognitive impairment). In relation to the interaction between opioids and constipation, it would have been important to check even for parallel dispensation of laxatives which we did not do. However, Christensen et al. [28] state that 48% of patients with opioid-induced constipation did not benefit from laxatives which argues in favour of our way of assessment.

A major challenge was the definition of time frames under which diseases should be coded. We differentiated between chronic diseases coded once during 5 years such as heart failure, “less chronic” diseases such as gastrointestinal bleeding coded once during 2 years, and ‘less severe’ diseases such as constipation coded twice during 2 years. This definition relied on unpublished sensitivity analyses and our clinical experience and may thus be subject to discussion. However, we think that neither of these limitations implied a substantial over- or underreporting of the true prevalence of DDSIs. Of note, we analysed only prescription drugs. As NSAIDs are available as over-the-counter drugs it is possible that the true prevalence of DDSIs is higher than described in our study.

Due to the cross-sectional design, it is not sure that the interacting disease preceded the dispensation of the interacting drug. Even an adverse drug reaction rather than a DDSI is possible: the use of NSAIDs lead to a peptic ulcer. This possibility cannot be ruled out, although it is unlikely; drug use was assessed during 2016, whereas interacting diseases were measured during 2012–2016 or 2015–2016. It is thus highly probable that the interacting disease was present before drug dispensation. A prospective approach, though more complicated, may give a clearer answer to those questions.

### Comparison with existing literature

We are not aware of a study that describes the prevalence of DDSIs in a European primary care setting using STOPP-START criteria version 2 [5]. A study from German primary care describes a prevalence of 10.4% using Beers Criteria [13] based on a patient group with predefined interacting diseases. This is in contrast to our study as we included all older patients in primary care. Regarding the prevalence of single DDSIs, Dreischulte et al. [29] found that 2.1% of older patients with heart failure in Scottish primary care used NSAIDs during a period of 8 weeks preceding the assessment date, whereas the corresponding percentage in our study was 7.9% (Appendix Table 4). A possible explanation of the threefold higher prevalence in our study is that we assessed NSAID use during one year, making it difficult to compare the findings. In general, it is important to note that DDSIs are less common than other types of potentially inappropriate prescribing such as drug-drug interactions [30] or excessive dosing in relation to impaired renal function [27].

Hanlon et al. analysed the prevalence of DDSIs by Beers Criteria in older adults living in a US community setting [12]. In keeping with findings in our study, NSAIDs were most frequently involved. The most prevalent DDSI with NSAID in Hanlon´s study was ‘peptic ulcer disease and aspirin/NSAIDs without gastroprotection’. However, Beers Criteria do not define hypertension/NSAID as a DDSI. The predominance of NSAIDs in our study relies mainly on the interaction between hypertension and NSAIDs. The clinical relevance of this DDSI may however be questioned: primary care patients with hypertension and regular NSAID use reached target blood pressure to the same extent as patients without NSAIDs [31]. This highlights the importance of prospective studies analysing to what extent DDSIs actually cause harm, as well as of regular updates of criteria of potentially inappropriate prescribing.

### Implications for research and/or practice

Physicians experience that potentially inappropriate prescribing is complex [32]. However, we found that only a limited number of drugs is implicated in the context of DDSIs. We analysed 31 DDSIs and found that NSAIDs were implicated in more than 80% of cases equal to five DDSIs. In view of the time constraints physicians in primary care face this finding may help to target medication reviews, and at the same time to increase their quality. Regarding DDSIs, GPs prescribing habits may successfully be improved if they focus on the inappropriate use of NSAIDs.

An interesting finding was that older patients with chronic pain and heart failure were prescribed NSAIDs to a significantly lower extent than those with chronic pain but without heart failure. A possible explanation may be that physicians are aware of DDSIs to a certain extent, and prescribe cautiously in older patients with multimorbidity. However, although we found up to 15% lower risk of NSAIDs in conjunction with heart failure, there is room for improvement of prescribing, as there were still 1,920 older patients with pain and heart failure who were dispensed NSAIDs at least once.

It is possible that this finding reflects physicians´ deliberate balancing of benefit against harm [33] rather than a more black or white concept of ‘potentially inappropriate prescribing’. Physician and patient may for instance have decided that the short-term use of NSAIDs against severe pain outweighs the possible aggravation of heart failure. Qualitative studies should elucidate to what extent physicians and older patients weight benefit and harm in shared decision-making. Moreover, there is an urgent need to implement multimorbidity guidelines [34] as an important supplement to the existing single-disease guidelines, allowing physicians to adjust drug use to the individual needs of older patients [6].

## Supplementary Material

Supplemental Material

## Additional information

This paper has an appendix including additional information.
