# Supplementary material for: Drug–disease interactions in Swedish senior primary care patients were dominated by non-steroid anti-inflammatory drugs and hypertension – a population-based registry study
Source: Scand J Prim Health Care. 2020 Jul 29;38(3):330–9. doi: 10.1080/02813432.2020.1794396 (PMC7470142; doi:10.1080/02813432.2020.1794396)
Supplement: Supplemental Material [file IPRI_A_1794396_SM2865.docx]

***Appendix to manuscript:***

**Drug-disease interactions in Swedish senior primary care patients were dominated by non-steroid anti-inflammatory drugs and hypertension - a population based registry study**

**Authors**

K Schmidt-Mende, M Andersen, B Wettermark, J Hasselström

**Affiliation**

K Schmidt-Mende, PhD student, Academic primary health care centre, Stockholm Region and Division of Family Medicine, Department of Neurobiology, Care Sciences and Society, Karolinska Institute, Sweden

Morten Andersen, professor, Department of Drug Design and Pharmacology, Faculty of Health and Medical Sciences, University of Copenhagen, Denmark

Björn Wettermark, professor, Department of Pharmacy, Uppsala University, Sweden

Jan Hasselström, MD PhD, Academic primary health care centre, Stockholm Region and Division of Family Medicine, Department of Neurobiology, Care Sciences and Society, Karolinska Institute, Sweden

**Corresponding author:**

Katharina Schmidt-Mende

e-mail: katharina.schmidt-mende@ki.se

telephone: 0046 – 737 - 75 41 78

Content

[Introduction 2](#_Toc31138295)

[Table 1: Selection of drug-disease interactions from 80 STOPP indicators 3](#_Toc31138296)

[Figure 1: Illustration of population in which prevalence of interacting drug was assessed 11](#_Toc31138297)

[Table 2: Prevalence of drug-disease interactions (DDSIs) in 336 295 individuals (figure 2a in manuscript) 12](#_Toc31138298)

[Table 3: Prevalence of interacting drugs in patients with an interacting disease (figure 2b in manuscript) 14](#_Toc31138299)

[Table 4: Prevalence differences and 95% confidence intervals of NSAIDs in patients with a pain diagnosis (n=101 925) and with/without an interacting disease (figure 3 in manuscript) 16](#_Toc31138300)

[References 17](#_Toc31138301)

# Introduction

We defined drug-disease interactions as “drugs that worsen a pre-existing condition” [1, 2].

Drug-disease interactions were chosen with reference to Irish STOPP Criteria version 2 [3]. STOPP/START criteria contain 114 indicators whereof 80 refer to misprescribing (STOPP criteria) and 34 to underprescribing (START criteria). The use of STOPP/START criteria in their entirety has been shown to improve clinical outcomes in older patients [26-28].

STOPP-START criteria do not include a specific list of drug-disease interactions, which is why we decided for each one of the 80 STOPP-indicators if it is a drug-disease interaction according to our definition (“drugs that worsen a pre-existing condition”) (table 1).

A total of 29 STOPP-criteria were drug-disease interactions. Of note, some of the indictors contained several (2-5) drug-disease interactions, for example D1 (“tricyclic antidepressants with dementia, narrow angle glaucoma, cardiac conduction abnormalities, prostatism, or prior history of urinary retention (risk of worsening these conditions)”). See table 1 and 2 for details.

Five STOPP-criteria that were drug-disease interactions (B2, B4, B13, D11, I1) could not be assessed by means of register data and were excluded.

Finally, 31 drug-disease interactions were assessed for their prevalence.

We defined a pain-diagnosis serving as indication for NSAID: ICD-10 diagnoses for osteoarthritis (M15-19) or rheumatologic disease (M05-14) ≥once 2012-16 or the same chronic pain diagnoses (pain in shoulder, leg, foot, back, fibromyalgia M75-77, M79, M53, M54, R52 without R52.0, migraine G43, headache G44, R51) ≥once 2015 and ≥once 2016.

# Table 1: Selection of drug-disease interactions from 80 STOPP indicators

| **Section/indicator** | **Reason why drug treatment is inappropriate** | **Comment** | **Included yes/no** |
| --- | --- | --- | --- |
| **Section A: Drug indication criteria** |  |  |  |
| A1. Any drug prescribed without an evidence-based clinical indication. | no indication |  | no |
| A2. Any drug prescribed beyond the recommended duration, where treatment duration is well defined. | too long duration |  | no |
| A3. Any duplicate drug class prescription e.g. two concurrent NSAIDs, SSRIs, loop diuretics, ACE inhibitors, anticoagulants (optimisation of monotherapy within a single drug class should be observed prior to considering a new agent). | duplication |  | no |
| **Section B: Cardiovascular System criteria** |  |  |  |
| B1. Digoxin for heart failure with preserved systolic ventricular function (no clear evidence of benefit) | no added benefit |  | no |
| B2. Verapamil or diltiazem with NYHA Class III or IV heart failure (may worsen heart failure). | drug-disease interaction | not measurable due to lack of clinical data | no |
| B3. Beta-blocker in combination with verapamil or diltiazem (risk of heart block). | drug-drug interaction |  | no |
| B4. Beta blocker with symptomatic bradycardia (< 50/min), type II heart block or complete heart block (risk of profound hypotension, asystole). | drug-disease interaction | bradycardia < 50/min not measurable due to lack of clinical data; presence of pacemaker not reliably assessable with ICD-10 diagnosis | no |
| B5. Amiodarone as first-line antiarrhythmic therapy in supraventricular tachyarrhythmias (higher risk of side-effects than beta-blockers, digoxin, verapamil or diltiazem) | Irrational choice, other drug treatment with less side effects available |  | no |
| B6. Loop diuretic as first-line treatment for hypertension (lack of outcome data for this indication; safer, more effective alternatives available). | Irrational choice, other drug treatment with less side effects available |  | no |
| B7. Loop diuretic for dependent ankle oedema without clinical, biochemical evidence or radiological evidence of heart failure, liver failure, nephrotic syndrome or renal failure (leg elevation and /or compression hosiery usually more appropriate). | Irrational choice, other drug treatment with less side effects available |  | no |
| B8. Thiazide diuretic with current significant hypokalaemia (i.e. serum K+ < 3.0 mmol/l), hyponatraemia (i.e. serum Na+ < 130 mmol/l) hypercalcaemia (i.e. corrected serum calcium > 2.65 mmol/l) or with a history of gout (hypokalaemia, hyponatraemia, hypercalcaemia and gout can be precipitated by thiazide diuretic) | drug-disease interaction | electrolyte disturbance is not a disease;  included as a drug-disease interaction on behalf of interaction between gout and thiazide | yes |
| B9. Loop diuretic for treatment of hypertension with concurrent urinary incontinence (may exacerbate incontinence). | drug-disease interaction |  | yes |
| B10. Centrally-acting antihypertensives (e.g. methyldopa, clonidine, moxonidine, rilmenidine, guanfacine), unless clear intolerance of, or lack of efficacy with, other classes of antihypertensives (centrally-active antihypertensives are generally less well tolerated by older people than younger people) | irrational choice, other drug treatment with less side effects available |  | no |
| B11. ACE inhibitors or Angiotensin Receptor Blockers in patients with hyperkalaemia. | risk for side effect | hyperkalemia is not a disease | no |
| B12. Aldosterone antagonists (e.g. spironolactone, eplerenone) with concurrent potassium-conserving drugs (e.g. ACEI’s, ARB’s, amiloride, triamterene) without monitoring of serum potassium (risk of dangerous hyperkalaemia i.e. > 6.0 mmol/l – serum K should be monitored regularly, i.e. at least every 6 months). | drug-drug interaction |  | no |
| B13. Phosphodiesterase type-5 inhibitors (e.g. sildenafil, tadalafil, vardenafil) in severe heart failure characterised by hypotension i.e. systolic BP < 90 mmHg, or concurrent daily nitrate therapy for angina (risk of cardiovascular collapse) | drug-disease interaction | not measurable due to lack of clinical data | no |
| **Section C: Coagulation System criteria** |  |  |  |
| C1. Long-term aspirin at doses greater than 160mg per day (increased risk of bleeding, no evidence for increased efficacy). | too long duration |  | no |
| C2. Aspirin with a past history of peptic ulcer disease without concomitant PPI (risk of recurrent peptic ulcer). | drug-disease interaction | - | yes |
| C3. Aspirin, clopidogrel, dipyridamole, vitamin K antagonists, direct thrombin inhibitors or factor Xa inhibitors with concurrent significant bleeding risk, i.e. uncontrolled severe hypertension, bleeding diathesis, recent non-trivial spontaneous bleeding) (high risk of bleeding). | risk for side effect |  | no |
| C4. Aspirin plus clopidogrel as secondary stroke prevention, unless the patient has a coronary stent(s) inserted in the previous 12 months or concurrent acute coronary syndrome or has a high grade symptomatic carotid arterial stenosis (no evidence of added benefit over clopidogrel monotherapy) | irrational choice, no added benefit |  | no |
| C5. Aspirin in combination with vitamin K antagonist, direct thrombin inhibitor or factor Xa inhibitors in patients with chronic atrial fibrillation without a clear indication for aspirin (no added benefit from aspirin) | no added benefit |  | no |
| C6. Antiplatelet agents with vitamin K antagonist, direct thrombin inhibitor or factor Xa inhibitors in patients with stable coronary, cerebrovascular or peripheral arterial disease without a clear indication for anticoagulant therapy (no added benefit from dual therapy). | no added benefit |  | no |
| C7. Ticlopidine in any circumstances (clopidogrel and prasugrel have similar efficacy, stronger evidence and fewer side-effects). | irrational choice, other drug treatment with less side effects available |  | no |
| C8. Vitamin K antagonist, direct thrombin inhibitor or factor Xa inhibitors for first deep venous thrombosis without continuing provoking risk factors for > 6 months, (no proven added benefit). | too long duration  no added benefit |  | no |
| C9. Vitamin K antagonist, direct thrombin inhibitor or factor Xa inhibitors for first pulmonary embolus without continuing provoking risk factors for > 12 months (no proven added benefit). | too long duration  no added benefit |  | no |
| C10. NSAID and vitamin K antagonist, direct thrombin inhibitor or factor Xa inhibitors in combination (risk of gastrointestinal bleeding). | drug-drug interaction |  | no |
| C11. NSAID with concurrent antiplatelet agent(s) without PPI prophylaxis (increased risk of peptic ulcer disease) | drug-drug interaction |  | no |
| **Section D: Central Nervous System criteria** |  |  |  |
| D1. Tricyclic antidepressants with dementia, narrow angle glaucoma, cardiac conduction abnormalities, prostatism, or prior history of urinary retention (risk of worsening these conditions). | drug-disease interaction | “cardiac conduction abnormalities” too vague, unclear what is meant; “history of urinary retention” is not a chronic disease | yes |
| D2. Initiation of tricyclic antidepressants as first-line antidepressant treatment (higher risk of adverse drug reactions with TCAs than with SSRIs or SNRIs). | irrational choice, other drug treatment with less side effects available |  | no |
| D3. Neuroleptics with moderate-marked antimuscarinic/anticholinergic effects (chlorpromazine, clozapine, flupenthixol, fluphenzine, pipothiazine, promazine, zuclopenthixol) with a history of prostatism or previous urinary retention (high risk of urinary retention). | drug-disease interaction | “history of urinary retention” is not a chronic disease;  chlorpromazine and pipothiazine are not available in Sweden | yes |
| D4. Selective serotonin re-uptake inhibitors (SSRI’s) with current or recent significant hyponatraemia i.e. serum Na+ < 130 mmol/l (risk of exacerbating or precipitating hyponatraemia). | risk for side effect | hyponatremia is not a disease | no |
| D5. Benzodiazepines for ≥ 4 weeks (no indication for longer treatment; risk of prolonged sedation, confusion, impaired balance, falls, road traffic accidents; all benzodiazepines should be withdrawn gradually if taken for > 2 weeks as there is a risk of causing a benzodiazepine withdrawal syndrome if stopped abruptly). | too long duration |  | no |
| D6. Antipsychotics (i.e. other than quetiapine or clozapine) in those with parkinsonism or Lewy Body Disease (risk of severe extra-pyramidal symptoms) | drug-disease interaction | Lewy Body disease very uncommon in primary care, not assessed | yes |
| D7. Anticholinergics/antimuscarinics to treat extra-pyramidal side-effects of neuroleptic medications (risk of anticholinergic toxicity) | prescribing cascade |  | no |
| D8. Anticholinergics/antimuscarinics in patients with delirium or dementia (risk of exacerbation of cognitive impairment). | drug-disease interaction | delirium excluded as it is not a chronic disease | yes |
| D9. Neuroleptic antipsychotic in patients with behavioural and psychological symptoms of dementia (BPSD) unless symptoms are severe and other treatments have failed (increased risk of stroke). | irrational drug choice | clinical data not available; could though be interpreted as drug-disease interaction: “in case of cardiovascular disease such as stroke higher risk for recurrence of stroke” | no |
| D10. Neuroleptics as hypnotics, unless sleep disorder is due to psychosis or dementia (risk of confusion, hypotension, extra-pyramidal side effects, falls). | irrational drug choice |  | no |
| D11. Acetylcholinesterase inhibitors with a known history of persistent bradycardia (< 60 beats/min.), heart block or recurrent unexplained syncope or concurrent treatment with drugs that reduce heart rate such as beta-blockers, digoxin, diltiazem, verapamil (risk of cardiac conduction failure, syncope and injury). | drug-disease interaction | recurrent syncope and bradycardia cannot reliably be assessed without clinical information; presence of pacemaker not reliably assessable with ICD-10 diagnosis | no |
| D12. Phenothiazines as first-line treatment, since safer and more efficacious alternatives exist (phenothiazines are sedative, have significant anti-muscarinic toxicity in older people, with the exception of prochlorperazine for nausea/vomiting/vertigo, chlorpromazine for relief of persistent hiccoughs and levomepromazine as an anti-emetic in palliative care). | irrational choice, other drug treatment with less side effects available |  | no |
| D13. Levodopa or dopamine agonists for benign essential tremor (no evidence of efficacy) | irrational choice |  | no |
| D14. First-generation antihistamines (safer, less toxic antihistamines now widely available). | irrational choice, other drug treatment with less side effects available |  | no |
| **Section E. Renal System criteria.** |  |  |  |
| E1. Digoxin at a long-term dose greater than 125µg/day if eGFR < 30 ml/min/1.73m2 (risk of digoxin toxicity if plasma levels not measured). | too high dose |  | no |
| E2. Direct thrombin inhibitors (e.g. dabigatran) if eGFR < 30 ml/min/1.73m2 (risk of bleeding) | risk for side effects | drugs do not deteriorate the underlying disease “impaired renal function” but rather cause a side effect | no |
| E3. Factor Xa inhibitors (e.g. rivaroxaban, apixaban) if eGFR < 15 ml/min/1.73m2 (risk of bleeding) | risk for side effects | drugs do not deteriorate the underlying disease “impaired renal function” but rather cause a side effect | no |
| E4. NSAID’s if eGFR < 50 ml/min/1.73m2 (risk of deterioration in renal function). | drug-disease interaction |  | yes |
| E5. Colchicine if eGFR < 10 ml/min/1.73m2 (risk of colchicine toxicity). | too high dose |  | no |
| E6. Metformin if eGFR < 30 ml/min/1.73m2 (risk of lactic acidosis). | risk for side effects |  | no |
| **Section F: Gastrointestinal System criteria.** |  |  |  |
| F1. Prochlorperazine or metoclopramide with Parkinsonism (risk of exacerbating Parkinsonian symptoms). | drug-disease interaction |  | yes |
| F2. PPI for uncomplicated peptic ulcer disease or erosive peptic oesophagitis at full therapeutic dosage for > 8 weeks (dose reduction or earlier discontinuation indicated). | too long duration |  | no |
| F3. Drugs likely to cause constipation (e.g. antimuscarinic/anticholinergic drugs, oral iron, opioids, verapamil, aluminium antacids) in patients with chronic constipation where non-constipating alternatives are appropriate (risk of exacerbation of constipation). | drug-disease interaction | “non-constipating alternatives are appropriate” cannot reliably be assessed without clinical information | yes |
| F4. Oral elemental iron doses greater than 200 mg daily (e.g. ferrous fumarate > 600 mg/day, ferrous sulphate > 600 mg/day, ferrous gluconate > 1800 mg/day; no evidence of enhanced iron absorption above these doses). | too high dose, no added benefit |  | no |
| **Section G. Respiratory System criteria.** |  |  |  |
| G1. Theophylline as monotherapy for COPD (safer, more effective alternative; risk of adverse effects due to narrow therapeutic index). | irrational choice, other drug treatment with less side effects available |  | no |
| G2. Systemic corticosteroids instead of inhaled corticosteroids for maintenance therapy in moderate-severe COPD (unnecessary exposure to long-term side-effects of systemic corticosteroids and effective inhaled therapies are available). | irrational choice, other drug treatment with less side effects available |  | no |
| G3. Anti-muscarinic bronchodilators (e.g. ipratropium, tiotropium) with a history of narrow angle glaucoma (may exacerbate glaucoma) or bladder outflow obstruction (may cause urinary retention). | drug-disease interaction | - | yes |
|  | drug-disease interaction |  | yes |
|  | drug-disease interaction |  | yes |
| G4. Benzodiazepines with acute or chronic respiratory failure i.e. pO2 < 8.0 kPa ± pCO2 > 6.5 kPa (risk of exacerbation of respiratory failure). | drug-disease interaction | chronic respiratory failure assessed as “obstructive sleep apnea syndrome” | yes |
| **Section H: Musculoskeletal System criteria.** |  |  |  |
| H1. Non-COX-2 selective non-steroidal anti-inflammatory drug (NSAID) with history of peptic ulcer disease or gastrointestinal bleeding, unless with concurrent PPI or H2 antagonist (risk of peptic ulcer relapse). | drug-disease interaction | - | yes |
| H2. NSAID with established hypertension (risk of exacerbation of hypertension) or heart failure (risk of exacerbation of heart failure). | drug-disease interaction | - | yes |
| H3. Long-term use of NSAID (>3 months) for symptom relief of osteoarthritis pain where paracetamol has not been tried (simple analgesics preferable and usually as effective for pain relief) | too long duration, irrational choice, other drug treatment with less side effects available |  | no |
| H4. Long-term corticosteroids (>3 months) as monotherapy for rheumatoid arthritis (risk of systemic corticosteroid side-effects). | too long duration |  | no |
| H5. Corticosteroids (other than periodic intra-articular injections for mono-articular pain) for osteoarthritis (risk of systemic corticosteroid side-effects). | too long duration, irrational choice, other drug treatment with less side effects available |  | no |
| H6. Long-term NSAID or colchicine for prevention of relapses of gout where there is no contraindication to a xanthine-oxidase inhibitor e.g. allopurinol, febuxostat (xanthine-oxidase inhibitors are first choice prophylactic drugs in gout). | too long duration, irrational choice, other drug treatment with less side effects available |  | no |
| H7. COX-2 selective NSAIDs with concurrent cardiovascular disease (increased risk of myocardial infarction and stroke) | drug-disease interaction |  | yes |
| H8. NSAID with concurrent corticosteroids without PPI prophylaxis (increased risk of peptic ulcer disease) | drug-drug interaction |  | no |
| H9. Oral bisphosphonates in patients with a history of upper gastrointestinal disease i.e. dysphagia, oesophagitis, gastritis, duodenitis, or peptic ulcer disease, or upper gastrointestinal bleeding (risk of relapse/exacerbation of oesophagitis, oesophageal ulcer, oesophageal stricture) | drug-disease interaction | - | yes |
| **Section I: Urogenital System criteria.** |  |  |  |
| I1. Antimuscarinic drugs for overactive bladder syndrome with concurrent dementia or chronic cognitive impairment (risk of increased confusion, agitation) or narrow-angle glaucoma (risk of acute exacerbation of glaucoma), or chronic prostatism (risk of urinary retention). | drug-disease interaction | - | yes |
|  | drug-disease interaction |  | yes |
|  | drug-disease interaction |  | yes |
| I2. Selective alpha-1 selective alpha blockers in those with symptomatic orthostatic hypotension or micturition syncope (risk of precipitating recurrent syncope) | drug-disease interaction? | diagnosis is not reliably assessable with an ICD-10 code | no |
| **Section J: Endocrine System criteria.** |  |  |  |
| J1. Sulphonylureas with a long duration of action (e.g. glibenclamide, chlorpropamide, glimepiride) with type 2 diabetes mellitus (risk of prolonged hypoglycaemia). | risk for side effects | drugs do not deteriorate the underlying disease diabetes but rather cause a side effect | no |
| J2. Thiazolidenediones (e.g. rosiglitazone, pioglitazone) in patients with documented heart failure (risk of exacerbation of heart failure) | drug-disease interaction |  | yes |
| J3. Beta-blockers in diabetes mellitus with frequent hypoglycaemic episodes (risk of suppressing hypoglycaemic symptoms). | risk for side effects |  | no |
| J4. Oestrogens with a history of breast cancer or venous thromboembolism (increased risk of recurrence). | drug-disease interaction |  | yes |
|  | drug-disease interaction |  | yes |
| J5. Oestrogens without progestogen in patients with intact uterus (risk of endometrial cancer). | risk for side effects |  | no |
| J6. Androgens in the absence of primary or secondary hypogonadism (risk of androgen toxicity; no proven benefit outside of hypogonadism indication). | risk for side effects  no added benefit |  | no |
| **Section K: Drugs that predictably increase the risk of falls in older people.** |  |  |  |
| K1. Benzodiazepines (sedative, may cause reduced sensorium, impair balance). | risk for side effects |  | no |
| K2. Neuroleptic drugs (may cause gait dyspraxia, Parkinsonism). | risk for side effects |  | no |
| K3. Vasodilator drugs (e.g. alpha-1 receptor blockers, calcium channel blockers, long-acting nitrates, ACE inhibitors, angiotensin I receptor blockers, diazoxide, minoxidil, hydralazine) with persistent postural hypotension i.e. recurrent drop in systolic blood pressure ≥ 20mmHg (risk of syncope, falls). | risk for side effects |  | no |
| K4. Hypnotic Z-drugs (e.g. zopiclone, zolpidem, zaleplon) (may cause protracted daytime sedation, ataxia). | risk for side effects |  | no |
| **Section L: Analgesic Drugs.** |  |  |  |
| L1. Use of oral or transdermal strong opioids (morphine, oxycodone, fentanyl, buprenorphine, diamorphine, methadone, tramadol, pethidine, pentazocine) as first line therapy for mild pain (WHO analgesic ladder not observed). | irrational choice, other drug treatment with less side effects available |  | no |
| L2. Use of regular (as distinct from PRN) opioids without concomitant laxative (risk of severe constipation). | irrational choice, other drug treatment with less side effects available | could though be interpreted as drug-disease interaction: “in case of pre-existing constipation higher risk for aggravation of constipation” | no |
| L3. Long-acting opioids without short-acting opioids for break-through pain (risk of non-control of severe pain) | irrational choice, other drug treatment with less side effects available |  | no |
| **Section M: Antimuscarinic/anticholinergic drug burden.** |  |  |  |
| M1: Concomitant use of two or more drugs with antimuscarinic/anticholinergic properties (e.g. bladder antispasmodics, intestinal antispasmodics, tricyclic antidepressants, first generation antihistamines) (risk of increased antimuscarinic/anticholinergic toxicity) | duplicates |  | no |

# Figure 1: Illustration of population in which prevalence of interacting drug was assessed

Illustration of study population (grey frame) and patient groups in which the prevalence of the interacting drug (non-steroidal anti-inflammatory drug, NSAID) was assessed. Example: drug-disease interaction between NSAID and heart failure


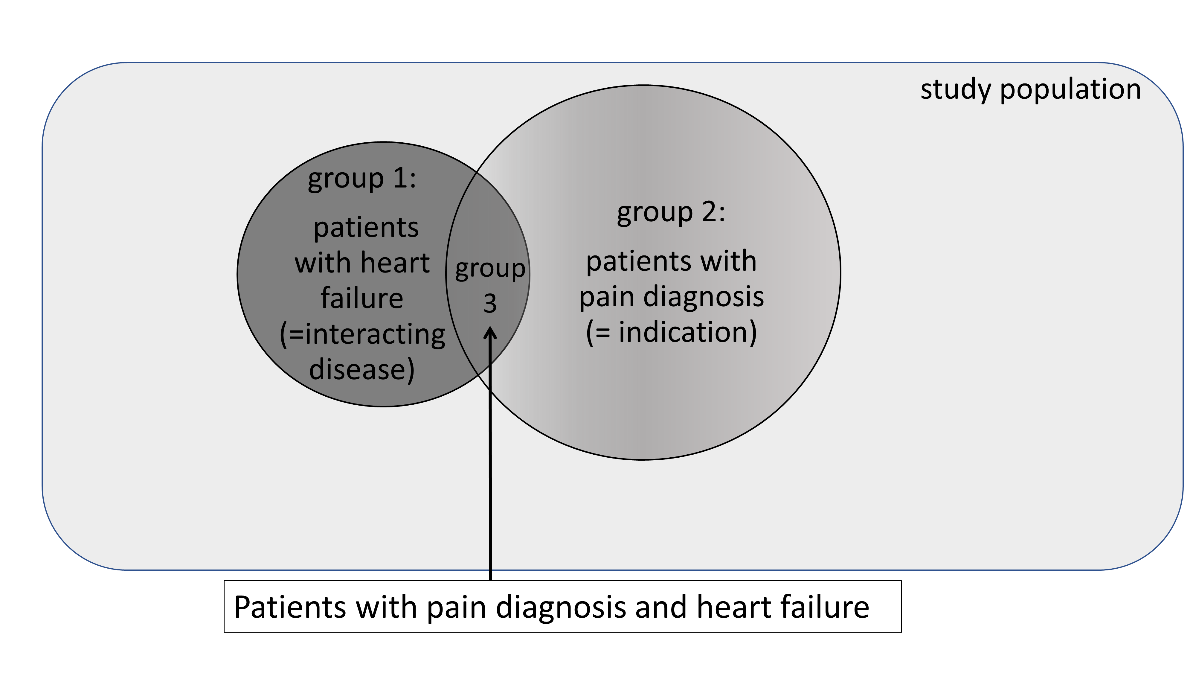


# Table 2: Prevalence of drug-disease interactions (DDSIs) in 336 295 individuals (figure 2a in manuscript)

| **Interacting drug** | **Interacting disease** | **Nr of patients with interacting disease** | **Nr of patients with DDSI** | **Prevalence of DDSI in total population (n=336 295)** |
| --- | --- | --- | --- | --- |
| NSAID | Hypertension | 179746 | 27128 | 8,07% |
| COX-2 selective NSAID | Concurrent cardiovascular disease^1^ | 62217 | 2402 | 0,71% |
| Loop diuretic | Urinary incontinence | 17647 | 2308 | 0,69% |
| NSAID | Heart failure | 24412 | 1920 | 0,57% |
| Antimuscarinic drugs | BPH | 24608 | 1814 | 0,54% |
| Anticholinergic drugs | Dementia | 16619 | 1655 | 0,49% |
| Antimuscarinic bronchodilators | BPH | 24608 | 1107 | 0,33% |
| NSAID | Impaired renal function | 13098 | 1049 | 0,31% |
| Antimuscarinic drugs | Dementia | 16619 | 749 | 0,22% |
| Thiazide diuretic | Gout | 12418 | 661 | 0,20% |
| Oral bisphosphonates | History of upper gastrointestinal disease^2^ | 13991 | 580 | 0,17% |
| Opioids | Constipation | 1564 | 533 | 0,16% |
| Benzodiazepines | Obstructive sleep apnea | 5665 | 528 | 0,16% |
| Low-dose acetylsalicylic acid without PPI | Peptic ulcer disease | 2193 | 428 | 0,13% |
| Tricyclic antidepressants | BPH | 24608 | 334 | 0,10% |
| Antimuscarinic/anticholinergic drugs | Constipation | 1564 | 327 | 0,10% |
| Tricyclic antidepressants | Dementia | 16619 | 253 | 0,08% |
| Non-COX-2 selective NSAIDs without PPI or H2-blockers | Peptic ulcer disease | 2193 | 168 | 0,05% |
| Oral iron | Constipation | 1564 | 139 | 0,04% |
| Neuroleptics | BPH | 24608 | 50 | 0,01% |
| Oestrogens | Breast cancer | 7099 | 35 | 0,01% |
| Prochlorperazine or metoclopramide | M Parkinson | 3239 | 28 | 0,01% |
| Antimuscarinic bronchodilators | Narrow angle glaucoma | 396 | 25 | 0,01% |
| Oestrogens | Venous thromboembolism | 6123 | 24 | 0,01% |
| Antipsychotics | M Parkinson | 3239 | 19 | 0,01% |
| Thiazolidenediones | Heart failure | 24412 | 16 | 0,00% |
| Antimuscarinic drugs | Narrow-angle glaucoma | 396 | 14 | 0,00% |
| Verapamil | Constipation | 1564 | 11 | 0,00% |
| Aluminium antacids | Constipation | 1564 | 10 | 0,00% |
| Tricyclic antidepressants | Narrow angle glaucoma | 396 | 8 | 0,00% |
| High-dose acetylsalicylic acid without proton-pump-inhibitor | Peptic ulcer disease | 2193 | 2 | 0,00% |

*BPH benign prostatic hyperplasia, NSAID non-steroidal anti-inflammatory drug, PPI proton-pump-inhibitor*

*^1^* *coronary heart disease, peripheral arterial disease, cerebrovascular disease, transient ischemic attack*

*^2^ i.e.* *dysphagia, oesophagitis, gastritis, duodenitis, or peptic ulcer disease, or upper gastrointestinal bleeding*

# Table 3: Prevalence of interacting drugs in patients with an interacting disease (figure 2b in manuscript)

| **Interacting disease** | **Nr of patients with interacting disease** | **Interacting drug** | **Nr of patients with interacting disease and interacting drug** | **Patients with interacting disease + interacting drug/patients with interacting disease in %** |
| --- | --- | --- | --- | --- |
| Hypertension | 179746 | NSAIDs | 27128 | 15.09% |
| Concurrent cardiovascular disease^1^ | 62217 | COX-2 selective NSAIDs | 2402 | 3.86% |
| BPH | 24608 | Antimuscarinic drugs | 1814 | 7.37% |
| BPH | 24608 | Anti-muscarinic bronchodilators | 1107 | 4.50% |
| BPH | 24608 | Tricyclic antidepressants | 334 | 1.36% |
| BPH | 24608 | Neuroleptics | 50 | 0.20% |
| Heart failure | 24412 | NSAIDs | 1920 | 7.86% |
| Heart failure | 24412 | Thiazolidenediones | 16 | 0.07% |
| Urinary incontinence | 17647 | Loop diuretic | 2308 | 13.08% |
| Dementia | 16619 | Anticholinergic drugs | 1655 | 9.96% |
| Dementia | 16619 | Antimuscarinic drugs | 749 | 4.51% |
| Dementia | 16619 | Tricyclic antidepressants | 253 | 1.52% |
| History of upper gastrointestinal disease^2^ | 13991 | Oral bisphosphonates | 580 | 4.15% |
| Impaired renal function | 13098 | NSAIDs | 1049 | 8.01% |
| Gout | 12418 | Thiazide diuretic | 661 | 5.32% |
| Breast cancer | 7099 | Oestrogens | 35 | 0.49% |
| Venous thromboembolism | 6123 | Oestrogens | 24 | 0.39% |
| Obstructive sleep apnea | 5665 | Benzodiazepines | 528 | 9.32% |
| M Parkinson | 3239 | Prochlorperazine or metoclopramide | 28 | 0.86% |
| M Parkinson | 3239 | Antipsychotics | 19 | 0.59% |
| Peptic ulcer disease | 2193 | Low-dose acetylsalicylic acid without | 428 | 19.52% |
| Peptic ulcer disease | 2193 | Non-COX-2 selective NSAIDs without PPI or H2-blockers | 168 | 7.66% |
| Peptic ulcer disease | 2193 | High-dose acetylsalicylic acid without PPI | 2 | 0.09% |
| Constipation | 1564 | Opioids | 533 | 34.08% |
| Constipation | 1564 | Antimuscarinic/anticholinergic drugs | 327 | 20.91% |
| Constipation | 1564 | Oral iron | 139 | 8.89% |
| Constipation | 1564 | Verapamil | 11 | 0.70% |
| Constipation | 1564 | Aluminium antacids | 10 | 0.64% |
| Narrow angle glaucoma | 396 | Anti-muscarinic bronchodilators | 25 | 6.31% |
| Narrow-angle glaucoma | 396 | Antimuscarinic drugs | 14 | 3.54% |
| Narrow angle glaucoma | 396 | Tricyclic antidepressants | 8 | 2.02% |

*BPH benign prostatic hyperplasia, NSAID non-steroidal anti-inflammatory drug, PPI proton-pump-inhibitor*

*^1^ coronary heart disease, peripheral arterial disease, cerebrovascular disease, transient ischemic attack*

*^2^ i.e. dysphagia, oesophagitis, gastritis, duodenitis, or peptic ulcer disease, or upper gastrointestinal bleeding*

# Table 4: Prevalence differences and 95% confidence intervals of NSAIDs in patients with a pain diagnosis (n=101 925) and with/without an interacting disease (figure 3 in manuscript)

| Column 1 | Column 2 | Column 3 | Column 4 | Column 5 | Column 6 | Column 7 | Column 8 | Column 9 |
| --- | --- | --- | --- | --- | --- | --- | --- | --- |
| **Interacting drug** | **Interacting disease** | **Prevalence difference^1^** | **Lower**  **95% CI** | **Upper**  **95% CI** | **Nr of patients in group 3^2^** | **Nr of patients in group 3^2^ with NSAID** | **Nr of patients in group 2^2^** | **Nr of patients in group 2^2^ with NSAID** |
| NSAIDs | Heart failure | -0.156 | -0.163 | -0.149 | 10 066 | 1 262 | 91 859 | 25 844 |
| NSAIDs | Impaired renal function | -0.15 | -0.159 | -0.141 | 5 720 | 711 | 96 205 | 26 395 |
| COX-2 selective NSAIDs | Concurrent cardiovascular disease^3^ | -0.041 | -0.045 | -0.037 | 21 640 | 1 511 | 80 285 | 8 932 |
| Non-COX-2 selective NSAIDs without PPI or H2-blockers | Peptic ulcer disease | -0.04 | -0.062 | -0.017 | 883 | 112 | 101 042 | 16 814 |
| NSAIDs | Hypertension | -0.028 | -0.034 | -0.022 | 62 509 | 15 948 | 39 416 | 11 158 |

*NSAID non-steroidal anti-inflammatory drug, PPI proton-pump-inhibitor*

*^1^prevalence difference=column 7/column 6 – column 9/column 8*

*^2^ see figure 1 in appendix*

*^3^ coronary heart disease, peripheral arterial disease, cerebrovascular disease, transient ischemic attack*

# References

1. Pugh MJ, Starner CI, Amuan ME, Berlowitz DR, Horton M, Marcum ZA, et al. Exposure to potentially harmful drug-disease interactions in older community-dwelling veterans based on the Healthcare Effectiveness Data and Information Set quality measure: who is at risk? J Am Geriatr Soc. 2011;59:1673-8.

2. Lindblad CI, Artz MB, Pieper CF, Sloane RJ, Hajjar ER, Ruby CM, et al. Potential drug-disease interactions in frail, hospitalized elderly veterans. Ann Pharmacother. 2005;39:412-7.

3. O'Mahony D, O'Sullivan D, Byrne S, O'Connor MN, Ryan C, Gallagher P. STOPP/START criteria for potentially inappropriate prescribing in older people: version 2. Age Ageing. 2015;44:213-8.
